# Security analysis of elliptic curves with embedding degree 1 proposed in PLOS ONE 2016

**DOI:** 10.1371/journal.pone.0212310

**Published:** 2019-02-19

**Authors:** Tadanori Teruya

**Affiliations:** Cyber Physical Security Research Center, National Institute of Advanced Industrial Science and Technology, Aomi, Koto-ku, Tokyo, Japan; Victoria University, AUSTRALIA

## Abstract

Wang et al. proposed a method for obtaining elliptic curves with embedding degree 1 for securing critical infrastructures, and presented several elliptic curves generated by their method with torsion points of 160 bits and 189 bits orders. They also presented some experimental results and claimed that their implementation of an elliptic curve generated with their method is faster than an implementation for embedded devices presented by Bertoni et al. In this paper, we point out that the security and efficiency claims given by Wang et al. are flawed. Specifically, we show that it is possible to solve finite field discrete logarithm problems defined over their elliptic curves in practice. On the elliptic curves with torsion points of 160 bits orders generated by Wang et al., their instances of finite field discrete logarithm problems are solved in around 4 hours by using a standard desktop PC. On the torsion points of 189 bits orders, their instances are solved in around 10 days by using two standard desktop PCs. The hardness of the finite field discrete logarithm problems is one of the most important bases of security; therefore, their elliptic curves should not be used for cryptographic purposes.

## Introduction

Since 2000, many researchers have proposed efficient and useful cryptographic schemes for securing systems using a *pairing*, which is a bilinear map defined over an elliptic curve. For example, Sakai et al. proposed a non-interactive key-exchange scheme [1], Joux proposed a tripartite key-exchange scheme [2], Boneh and Franklin proposed an identity-based encryption scheme [3], Boneh et al. proposed a short digital signature scheme [4], and Groth and Sahai proposed efficient non-interactive zero-knowledge proof systems [5]. This research field is called *pairing-based cryptography* because a pairing is used as a building block. As mentioned above, the pairings allow us to implement many efficient and useful cryptographic schemes, and pairing-based cryptography is currently one of the major fields of cryptographic research [6, 7].

For security requirements, it is necessary for any implementation of a pairing-based cryptographic scheme to appropriately select an elliptic curve. Its efficiency should then be improved for practical performance requirements. Since the computational costs of pairings and group operations defined over elliptic curves are expensive, investigation of fast algorithms and implementations is an important research topic, and there have been many studies on the mathematical foundation for algorithms [8–26], and efficient implementations [27–34]. A comprehensive survey was presented by [7].

Thanks to these studies, the development of pairing-based cryptosystems has progressed in not only theory but also practice. However, implementation is still difficult. The most difficult problem is the selection of appropriate parameters to instantiate the schemes securely and efficiently. This is because all algorithms correctly work, even if a selected parameter is vulnerable. It is necessary to carefully evaluate whether the selected parameters are robust against cryptanalysis.

Recently, Wang et al. [35] proposed a method for obtaining elliptic curves with embedding degree 1 in order to instantiate pairing-based cryptographic schemes for securing critical infrastructures. They presented some experimental results and claimed that their pairing implementation of an elliptic curve generated with their method is faster than another secure and efficient implementation presented by Bertoni et al. [36, 37]. The aim of Bertoni et al. [36, 37] is to develop efficient implementation of pairings for embedded devices.

In this paper, we point out that there is a serious issue with the security and efficiency claims given by Wang et al. [35]. In short, their elliptic curves are insecure and should not be used for cryptographic purposes. Wang et al. [35] presented several elliptic curves generated by their method. The bit lengths of orders of these curves are 160 bits and 189 bits, and these elliptic curves are described in the files [38, 39] of their supporting information. According to [25, 40, 41], these bit lengths are large enough to guarantee security in practice. However, we demonstrate that finite field discrete logarithm problems defined on these elliptic curves are solvable in practice by using standard desktop PCs. The hardness of finite field discrete logarithm problems is one of the most important bases of security; therefore, their elliptic curves should not be used for cryptographic purposes. In addition, we present security and efficiency analyses of the method of Wang et al. [35]. Based on these analyses, we conclude that the claims of security and efficiency given by Wang et al. [35] are flawed.

Recently, Chatterjee et al. [24] proposed proper constructions of pairing-based cryptographic schemes on elliptic curves with embedding degree 1. Their considerations and analyses of security and efficiency of their constructions are comprehensively discussed based on state-of-the-art results; therefore, we do not discuss how to repair the method of Wang et al. [35] in this paper. We strongly recommend that the readers refer to the study by Chatterjee et al. [24].

The rest of this paper is organized as follows. We introduce preliminaries of pairing-based cryptography. Next, we also introduce finite field discrete logarithm problems and elliptic curve discrete logarithm problems in pairing-based cryptography. We show how to produce finite field discrete logarithm problems from elliptic curves generated by Wang et al. [35], and then we show that they are solvable by using standard desktop PCs. Then we explain why their claims are flawed. Finally, we conclude the paper.

## Materials and methods

### Mathematical preliminaries of pairings

In this section, we introduce the notations and terminology of pairings defined over elliptic curves [6, 7].

Let *S* be a finite set, then we denote the number of elements in *S* by #*S*. Let *p* be a prime number, and *k* be a positive integer. We denote by $F_{p}$ a finite field whose field order is *p*, by $F_{p^{k}}$ its *k*-th extension field, and by $F_{p^{k}}^{*}$ the multiplicative group of $F_{p^{k}}$. For $F_{p^{k}}$, its characteristic and extension degree are *p* and *k*, respectively. We assume that *p* > 3, and an elliptic curve *E* defined over $F_{p}$ is defined by the Weierstrass equation

$$\begin{array}{c} Y^{2}=X^{3}+aX+b, \end{array}$$ (1)

where *X* and *Y* are two variables, and $a,b\in F_{p}$ with 4*a*^3^ + 27*b*^2^ ≠ 0. We define the $F_{p}$-rational points of *E* as

$$\begin{array}{c} E\left(F_{p}\right)≔\left\{\left(x,y\right)\in F_{p}\times F_{p}|y^{2}=x^{3}+ax+b\right\}\cup \left\{\infty \right\}, \end{array}$$ (2)

where ∞ is the point at infinity. Note that $E(F_{p})$ and its group operation, which is denoted by addition symbol “+” in this paper, forms an abelian group whose unit element is ∞ and inverse operation is −(*x*, *y*) ≔ (*x*, −*y*). Let $P\in E(F_{p})$ and let *a* be an integer. We denote the scalar multiplication of *P* by *a* as

$$\begin{array}{c} \left[a\right]P≔\underset{a\;\text{repetitions}}{\underset{︸}{P+P+\cdots +P}} \end{array}$$ (3)

if *a* > 0, [*a*]*P* ≔ [−*a*](−*P*) if *a* < 0, and [0]*P* ≔ ∞. We say that *P* is order *n* if *n* is the smallest positive integer such that [*n*]*P* = ∞. We also denote an additive cyclic group generated by *P* as

$$\begin{array}{c} \langle P\rangle ≔\{Q|\text{there}\;\text{exists}\;a\in Z\;\text{such}\;\text{that}\;Q=[a]P\}, \end{array}$$ (4)

and a multiplicative cyclic group generated by *x* as

$$\begin{array}{c} \left\langle x\right\rangle ≔\left\{y|\text{there}\;\text{exists}\;a\in Z\;\text{such}\;\text{that}\;y=x^{a}\right\}. \end{array}$$ (5)

We call *P* and *x* are generators of 〈*P*〉 and 〈*x*〉, respectively. We also call the number of elements of a group the group order.

Let *r* be a prime number with *r* ≠ *p*. Then we define the *r*-torsion points as

$$\begin{array}{c} E\left[r\right]≔\{P\in E\left({\bar{F}}_{p}\right)|\left[r\right]P=\infty \}, \end{array}$$ (6)

where ${\bar{F}}_{p}$ is the algebraic closure of $F_{p}$, and we call *r* the order of *E*[*r*]. The *embedding degree k* of *E* with respect to *r* is the smallest positive integer such that *r*∣(*p*^*k*^ − 1), and this property ensures $\mu_{r}\subseteq F_{p^{k}}^{*}$ and $E\left[r\right]\subseteq E\left(F_{p^{k}}\right)$, where $\mu_{r}≔\left\{x\in F_{p^{k}}^{*}|x^{r}=1\right\}$ is the *r*-th roots of unity [42]. Note that *μ*_*r*_ = 〈*x*〉 for all *x* ∈ *μ*_*r*_\{1} if *r* is prime.

Let *P*, *Q* ∈ *E*[*r*], then we define the *reduced Tate pairing* *t*: *E*[*r*] × *E*[*r*] → *μ_r_*. Note that the reduced Tate pairing *t* has the following three properties: First, it is non-degenerate, i.e., for all elements *P* ∈ *E*[*r*]\{∞}, there is an element *Q* ∈ *E*[*r*] with *e*(*P*, *Q*) ≠ 1, and for all elements *Q* ∈ *E*[*r*]\{∞}, there is an element *P* ∈ *E*[*r*] with *e*(*P*, *Q*) ≠ 1. Second, it is bilinear, i.e., for all *P*, *Q*, *S* ∈ *E*[*r*], *t*(*P* + *S*, *Q*) = *t*(*P*, *Q*) · *t*(*S*, *Q*) and *t*(*P*, *Q* + *S*) = *t*(*P*, *Q*) · *t*(*P*, *S*). Third, it is efficiently computable, i.e., its computational time complexity is in the polynomial of log *r* (see [8, 9]).

### Discrete logarithm problems of pairing-based cryptography

Every pairing-based cryptographic scheme requires the hardness of underlying mathematical problems in order to guarantee security in practice. In this section, we introduce two discrete logarithm problems which are important underlying mathematical problems for pairing-based cryptographic schemes.

Let *E* be an elliptic curve defined over a finite field $F_{p}$, let *r* be a prime number with *r* ≠ *p*, and let *k* be the embedding degree of *E* with respect to *r*. We define the following two discrete logarithm problems of *E*:

- *Elliptic curve discrete logarithm problem in E[r] (ECDLP)*: Given *P* ∈ *E*[*r*]\{∞} and *Q* = [*x*]*P*, where *x* is a randomly chosen integer from {1, …, *r* − 1}, find *x*. We call a pair of *P* and *Q* an instance of ECDLP, and denote its solution by *x* = log_*P*_ *Q*.
- *Finite field discrete logarithm problem in $\mu_{r}\subseteq F_{p^{k}}^{*}$ (FFDLP)*: Given *g* ∈ *μ*_*r*_\{1} and *h* = *g*^*y*^, where *y* is a randomly chosen integer from {1, …, *r* − 1}, find *y*. We call a pair of *g* and *h* an instance of FFDLP, and denote its solution by *y* = log_*g*_ *h*.

In general, the hardness of solving ECDLP is determined by the robustness against the best solving algorithm for ECDLP. The computational time complexity of the algorithm strongly depends on the bit length of order *r*, and the larger the bit length of *r* is, the harder ECDLP becomes. On the other hand, the hardness of solving FFDLP is also determined by the robustness against the best solving algorithm for FFDLP. Also, the computational time complexity of the algorithm strongly depends on the bit length of field order *p*^*k*^, and the larger the bit length of *p*^*k*^ is, the harder FFDLP becomes.

Note that Menezes et al. [43], and Frey and Rück [44] pointed out that ECDLP is reduced to FFDLP by using the Weil pairing or the (reduced) Tate pairing. We call this reduction the *pairing reduction*. For example, given an instance of ECDLP *P* and *Q* = [*x*]*P* as above, one can choose an arbitrary element *T* ∈ *E*[*r*] with *t*(*P*, *T*) ≠ 1 then construct an FFDLP instance *g* ≔ *t*(*P*, *T*) and *h* ≔ *t*(*Q*, *T*) = *t*([*x*]*P*, *T*) = *t*(*P*, *T*)^*x*^; thus, log_*P*_ *Q* = log_*g*_ *h*. As mentioned above, every cryptographic scheme requires the hardness of ECDLP and FFDLP. Here it is important to note that *the overall hardness of every cryptographic scheme is determined by the weakest underlying problem*. Hence, developers of pairing-based cryptosystems should use appropriate elliptic curves such that *both ECDLP and FFDLP are intractable simultaneously*.

As mentioned above, the longer the bit lengths of *r* and *p*^*k*^ are, the harder ECDLP and FFDLP, respectively, become and imply stronger robustness against cryptanalysis. However, the longer bit lengths of *r* and *p*^*k*^ cause significant efficiency loss. Hence, it is a very important task to find an elliptic curve which has *r* and *p*^*k*^ achieving reasonable robustness (i.e., security) and efficiency simultaneously. Since we focus on solving FFDLP reduced from ECDLP of elliptic curves generated by Wang et al. [35], finding appropriate elliptic curves is out of scope of this paper, and we refer the reader to [25, 40, 45, 46] for the details.

### Elliptic curves with embedding degree 1 proposed in PLOS ONE 2016

Wang et al. [35] presented two types of elliptic curves with embedding degree 1 generated by their method. These are described in the files [38, 39] of their supporting information. For each type, there are 10 elliptic curves. Hence, there are 20 elliptic curves in total.

In this paper, we denote these two types of elliptic curves [38] and [39] as W160 and W189, respectively. In W160, each elliptic curve *E* is defined over a finite field $F_{p_{1}}$ with $E\left[r_{1}\right]\subseteq E\left(F_{p_{1}}\right)$, where *p*_1_ and *r*_1_ are two distinct prime numbers. The bit lengths of *r*_1_ and *p*_1_ are 160 bits and 319 bits, respectively. In W189, each elliptic curve *E* is defined over a finite field $F_{p_{2}}$ with $E\left[r_{2}\right]\subseteq E\left(F_{p_{2}}\right)$, where *p*_2_ and *r*_2_ are two distinct prime numbers. The bit lengths of *r*_2_ and *p*_2_ are 189 bits and 377 bits, respectively.

## Results

In this section, we demonstrate that the FFDLP instances reduced from ECDLP instances of elliptic curves generated by Wang et al. [35] are easily solvable. Wang et al. [35] claimed that implementations of pairings defined over these elliptic curves are more efficient than an implementation of Bertoni et al. [36, 37]. To the best of our knowledge, ECDLP and FFDLP of an elliptic curve implemented by Bertoni et al. [36, 37] are intractable, so that we naturally expected that ECDLP and FFDLP of the elliptic curves implemented by Wang et al. [35] are also intractable. However, this is not the case, and hence, the security claim given by Wang et al. [35] is clearly flawed, and the efficiency claim is also flawed because FFDLP instances reduced from ECDLP instances of their elliptic curves are solvable in practice.

### Problem instance generation

In this section, we explain how we generate the FFDLP instances reduced from ECDLP instances, which we solve.

To demonstrate that FFDLP instances are easily solvable in the W160 and W189 elliptic curves, the FFDLP instances should be generated without knowing their solutions. However, recall that the definition of FFDLP is that its solution is randomly chosen by an instance generator, and this means that the instance generator knows the solution. To generate FFDLP instances without knowing their solutions, we use well-known methodology [47–50] and the pairing reduction [43, 44].

Concretely, we generate *h*, which is a part of FFDLP instances, by using the ratio *π* of a circle’s circumference to its diameter. For an elliptic curve *E* defined over an finite field $F_{p}$, we compute *h* ≔ (⌊*π* · 2^*ℓ*^⌋)^*c*^ mod *p*, where *ℓ* is the largest integer such that ⌊*π* ⋅ 2^*ℓ*^⌋ < *p*, and *c* = (*p* − 1)/*r*. Note that *h* ∈ *μ*_*r*_. For the W160 and W189 elliptic curves, we find the following two largest integers ⌊*π* ⋅ 2^316^⌋ and ⌊*π* ⋅ 2^374^⌋, respectively, that are less than *p*_1_ and *p*_2_, respectively. We use $h_{1}≔{\left(\left\lfloor \pi \cdot 2^{316}\right\rfloor \right)}^{c_{1}}\;\mathrm{mod}\;p_{1}$ and $h_{2}≔{\left(\left\lfloor \pi \cdot 2^{374}\right\rfloor \right)}^{c_{2}}\;\mathrm{mod}\;p_{2}$ for W160 and W189, respectively, where *c*_1_ = (*p*_1_ − 1)/*r*_1_ and *c*_2_ = (*p*_2_ − 1)/*r*_2_, respectively. The integer values of ⌊*π* ⋅ 2^316^⌋, *h*_1_, ⌊*π* ⋅ 2^374^⌋, and *h*_2_ are described in files S1 and S2 Files of our supporting information.

Next, Wang et al. [35] provided elements *P*, *Q* ∈ *E*[*r*] for all the elliptic curves, so there are 20 pairs of *P*, *Q*. We use them to generate 20 elements *g* = *t*(*P*, *Q*) for the remaining part of FFDLP instances. Note that, to compute *t*(*P*, *Q*), we use a function of the reduced Tate pairing implemented in Sage [51].

### Equipment

We explain our equipment to solve FFDLP instances. To solve FFDLP instances reduced from ECDLP instances of the W160 elliptic curves, we use one desktop PC with a Core i7-6700 (3.4) CPU. For the W189 elliptic curves, we use two desktop PCs with Core i7-6700 (3.4) and Core i7-4770 (3.4) CPUs. In these PCs, the size of equipped random access memories is 32, and the operating system is Ubuntu 16.04. We use the mathematics software system Sage [51] version 7.6 to generate FFDLP instances, the FFDLP solver CADO-NFS [52] version 2.3.0-rc1, and compiler gcc version 5.4.0.

### Solutions and calculation times of finite field discrete logarithm problems

We solve the FFDLP instances defined above. For the W160 and W189 elliptic curves, calculation times to obtain solutions are around 3 hours 45 minutes and 240 hours 4 minutes, respectively. Lists of FFDLP instances and their solutions are shown in S1 and S2 Files of our supporting information. Additionally, we present a verification script in S3 File.

According to the results of our demonstration, we conclude that the security and efficiency claims given by Wang et al. [35] are flawed, and all the elliptic curves described in the files [38, 39] of their supporting information should not be used for cryptographic purposes.

## Discussion

We have already shown that the FFDLP instances reduced from ECDLP instances of all the elliptic curves generated by Wang et al. [35] are practically solvable. In this section, we discuss why the security and efficiency claims given by Wang et al. [35] are flawed.

Wang et al. [35] presented the performance comparison of the reduced Tate pairing between their implementation of a W160 elliptic curve [38] and an implementation presented by Bertoni et al. [36]. Wang et al. [35] claimed that their implementation is faster than the latter. To revisit this comparison, we briefly introduce the implementation for embedded devices presented by Bertoni et al. [36]. Bertoni et al. [36, 37] implemented a pairing defined over another elliptic curve whose embedding degree is 2, and this elliptic curve is defined over $F_{p}$, where the bit length of *p* is 512 bits. We denote this elliptic curve as B160. Now, we show the bit lengths of orders *r* and field orders *p*^*k*^ of the W160, W189, and B160 elliptic curves in Table 1.

**Table 1:** Bit lengths of orders and field orders of elliptic curves proposed by Wang et al. and Bertoni et al.

	Bit length of *r* (bit)	Bit length of *p*^*k*^ (bit)
**W160**	160	319
**W189**	189	377
**B160**	160	1023

We consider the hardness of ECDLP and FFDLP of these elliptic curves based on state-of-the-art reports of hardness evaluation.

For ECDLP, Bernstein et al. [41] gave a state-of-the-art report on December 2nd 2016. They solved an ECDLP instance, where the bit length of order *r* is 117.35 bits. In general, the hardness of ECDLP strongly depends on the bit length of *r*. Therefore, solving ECDLP instances should be considered feasible if the bit length of *r* is less than 117.35 bits.

On the other hand, according to Grémy and Guillevic [53], state-of-the-art reports for solving FFDLP were given by Adrian et al. [54], Kleinjung et al. [50], Fried et al. [55], and Barbulescu et al. [49] on May 20th 2015, June 16th 2016, October 10th 2016, and April 29th 2015, respectively, and they solved FFDLP instances over finite fields $F_{p}^{*}$ and $F_{p^{2}}^{*}$, where the bit lengths of their field orders are 512 bits, 768 bits, 1024 bits, and 595 bits, respectively. In general, the hardness of FFDLP strongly depends on the bit length of *p*^*k*^. Fried et al. [55] solved the largest bit length of FFDLP; however, their technique to solve FFDLP is only applicable to certain finite fields that have a special property, and thus this result is not relevant for the discussion of the hardness of W160, W189, and B160 elliptic curves (and to the best of our knowledge, it seems to be not satisfied by these curves). Therefore, for embedding degrees *k* = 1 and 2, solving FFDLP instances should be considered feasible if the bit length of *p*^*k*^ is less than 768 bits. It is clear that FFDLP instances of the W160 and W189 elliptic curves [38, 39] are practically solvable, but instances of B160 are not. In fact, we solved the FFDLP instances of the W160 and W189 elliptic curves [38, 39], and this immediately implies that the performance comparison given by Wang et al. [35] is definitely unfair.

For a fair comparison, both ECDLP and FFDLP should be intractable, and the hardness of both ECDLP and FFDLP should ideally be equal. To the best of our knowledge, using the method of Wang et al. [35] to achieve the same hardness with B160, the bit lengths of *r* and *p* should be greater than or equal to at least 160 bits and 1023 bits, respectively, and the resulting elliptic curve should have almost the same hardness of FFDLP as a B160 elliptic curve [36, 37]. However, their method seems to output an elliptic curve, where the bit length of the resulting *p* is twice that of the resulting *r*. Therefore, the bit length of *r* should be around 512 bits, and this is larger than for the B160 elliptic curve [36, 37]. Both ECDLP and FFDLP defined over these elliptic curves should be intractable, and the hardness of FFDLP is almost the same because the bit length of *p*^*k*^ is almost the same. However, the resulting elliptic curve is defined over $F_{p}$, where the bit length of *p* is around 1023 bits and this is larger than that of B160 (recall that the B160 elliptic curve is defined over $F_{p}$, where the bit length of *p* is 512 bits, because its embedding degree is 2). In fact, larger bit lengths of *p* and *r* generally cause efficiency loss. Therefore, in the *same security level*, the implementation of B160 [36, 37] should be faster than implementations of elliptic curves generate by the method of Wang et al. [35].

From the discussions and considerations above, we conclude that there is no merit of the method proposed by Wang et al. [35].

## Conclusion

In this paper, we demonstrated that instances of finite field discrete logarithm problems derived from elliptic curves [38, 39] are solvable in practice. These elliptic curves are generated by the method of Wang et al. [35]. In our demonstration, the instances were generated without knowing their solutions, and they were solved by using standard desktop PCs in reasonable time. The hardness of discrete logarithm problems is one of the most important bases of security; therefore, the elliptic curves described in [35, 38, 39] should not be used for cryptographic purposes. We also pointed out that the efficiency evaluation given by Wang et al. [35] is unfair. From our demonstrations and discussions, it is clear that the security and efficiency claims given by Wang et al. [35] are flawed.

Finally, we recommend that the readers refer to the paper by Chatterjee et al. [24]. They presented careful and comprehensive discussions and proper constructions of pairing-based cryptographic schemes on elliptic curves whose embedding degree is 1, based on state-of-the-art results.

## Supporting information

S1 FileList of inputs and solutions of FFDLP instances of 160 bits (W160) elliptic curves.This file contains five integers *r*_1_, *p*_1_, *ℓ*_1_, *s*_1_, and *h*_1_, and ten sets of values *b*, *P*, *Q*, *g*, and *y*, and these symbols are defined as follows:
*r*_1_ is the order of each $E\left[r_{1}\right]\subseteq E\left(F_{p_{1}}\right)$.*p*_1_ is the field order of defining the finite field $F_{p_{1}}$ of each *E*.*ℓ*_1_ = 316.$s_{1}=\left\lfloor \pi \cdot 2^{\ell_{1}}\right\rfloor$, where *π* is the ratio of a circle’s circumference to its diameter.
$h_{1}=s_{1}^{c}\in \mu_{r_{1}}$, where *c* = (*p*_1_ − 1)/*r*_1_.*b* is a coefficient of each Weierstrass equation, which defines each *E*.*P* and *Q* are contained in each *E*[*r*_1_].*g* = *t*(*P*, *Q*).*y* = log_*g*_ *h*_1_.
Note that all *p*_1_, *r*_1_, *b*, *P*, and *Q* are generated by Wang et al. [35] and are described in the file [38].(TXT)

S2 FileList of inputs and solutions of FFDLP instances of 189 bits (W189) elliptic curves.This file contains five integers *r*_2_, *p*_2_, *ℓ*_2_, *s*_2_, and *h*_2_, and ten sets of values *b*, *P*, *Q*, *g*, and *y*, and these symbols are defined as follows:
*r*_2_ is the order of each $E\left[r_{2}\right]\subseteq E\left(F_{p_{2}}\right)$.*p*_2_ is the field order of defining the finite field $F_{p_{2}}$ of each *E*.*ℓ*_2_ = 374.
$s_{2}=\left\lfloor \pi \cdot 2^{\ell_{2}}\right\rfloor$, where *π* is the ratio of a circle’s circumference to its diameter.
$h_{2}=s_{2}^{c}\in \mu_{r_{2}}$, where *c* = (*p*_2_ − 1)/*r*_2_.*b* is a coefficient of each Weierstrass equation, which defines each *E*.*P* and *Q* are contained in each *E*[*r*_2_].*g* = *t*(*P*, *Q*).*y* = log_*g*_ *h*_2_.
Note that all *p*_2_, *r*_2_, *b*, *P*, and *Q* are generated by Wang et al. [35] and are described in the file [39].(TXT)

S3 File Sage script for verification. This Sage script verifies our results described in S1 and S2 Files.
(SAGE)
